# A Look Into the Next Century After 100 Years of Insulin

**DOI:** 10.7759/cureus.30133

**Published:** 2022-10-10

**Authors:** Sonal Gupta, Sourya Acharya, Samarth Shukla

**Affiliations:** 1 Department of Medicine, Jawaharlal Nehru Medical College, Datta Meghe Institute of Medical Sciences (Deemed to be University), Wardha, IND; 2 Department of Pathology, Jawaharlal Nehru Medical College, Datta Meghe Institute of Medical Sciences (Deemed to be University), Wardha, IND

**Keywords:** jet injectors, smart insulin, inceptor, insulin signaling, intelligent capsules

## Abstract

Being diagnosed with diabetes can be challenging, particularly if we do not know a similar individual with the same disease. A person's life may suddenly change and he/she may not be aware of the possibilities or treatment options available or the questions that need to be asked; hence, instead of looking for assistance, many people hide their diabetic condition from others. But due to innovative devices, individuals no longer need to be concerned. Various gadgets today help provision insulin via a subcutaneous route, for example, an insulin pen, pump, vial, or syringe. Despite being the most familiar way to provide insulin, subcutaneous insulin delivery is linked to needle pain, injection anxiety, lipodystrophy, compliance issues, and peripheral hyperinsulinemia; thus, there is a need for an insulin delivery system that is both less invasive and more biological. The discovery of insulin and its uses are linked to the beginning of diabetes treatment. Initially, the delivery of insulin was accomplished using giant, heavy, reusable syringes equipped with plungers, barrels, and wide-bore needles. To prepare these syringes and needles for reuse, these were boiled. The development of insulin syringes, which are presently in use and have revolutionized healthcare, resulted from manufacturers' continued efforts and technological innovations. Injections of insulin may become obsolete if research from the University of Alberta is successful. Researchers from the University of Alberta claim that insulin-producing cells developed from stem cells are secure for transplantation to wean diabetes patients from injectable insulin permanently. In a significant mice experiment, experts demonstrated the role of the inceptor (insulin inhibitory receptor), which protects the beta cells from insulin pathway activation. Insulin resistance in diabetes may be exacerbated by the inceptor's ability to block insulin signaling. Technologies known as "smart insulin" (glucose-responsive insulin) deliver insulin according to the patient's glycemic condition without needing additional monitoring by the patient or the physician in charge. The review of insulin administration devices and several cutting-edge insulin-related ideas are the main topics of this article.

## Introduction and background

A metabolic condition with numerous etiological factors is diabetes mellitus. High blood sugar and chronic aberrations in the carbohydrate, lipid, and protein metabolism are its defining characteristics. These defects in insulin action or release, or occasionally both, are to be blamed. [1]. They are linked to the emergence of the unique microvascular consequences of retinopathy, including neuropathy, nephropathy, and kidney failure, which can result in blindness [2]. The latter entails the danger of autonomic neural malfunction, foot ulceration, and amputation. A heightened incidence of the macrovascular disease is also linked to diabetes. Thirst, frequent urination, blurred eyesight, and losing weight are typical clinical manifestations, which may result in hyperosmolar nonketotic coma or ketoacidosis. Generally, presentations are minimal or nonexistent, and minor hyperglycemia can last for years while causing tissue damage, even when a person is symptom-free [3].

Diabetes mellitus affects a comparatively substantial portion of the global population. Type II diabetes accounts for 90% of patients, and type I diabetes accounts for 5-10% of the total cases. For patients with type I diabetes mellitus, providing Insulin is crucial; however, type II diabetic patients may administer it in the later stages [4]. Insulin, its delivery, and its future are the subjects of this review. Before prescribing, distributing, or administering insulin, a clinical diagnosis of hyperglycemia should be verified. For all patients having type I diabetes mellitus, insulin is the first-line treatment [5]. The main varieties of insulin therapy are long, ultra-long, intermediate-acting insulin, and rapid or short-acting insulin [6]. The differences in types of insulin are given in Table 1.

**Table 1:** Differences between various varieties of insulin therapy [7]

Type	Onset	Peak	Duration	Particulars
Rapid-acting analog of insulin	5-15 minutes	30-60 minutes	2-5 hours	It can be administered at the beginning of a meal.
Short-acting (soluble/regular insulin)	30 minutes	1-3 hours	4-8 hours	Inject it 15-30 minutes prior to a meal.
Intermediate or long-acting insulin (isophane or zinc insulin)	1-2 hours (NPH, Lente), 2-3 hours (Ultralente)	4-8 hours, 4-8 hours	8-12 hours (NPH), 8-24 hours (Ultralente)	It controls glycemic levels between meals and can be given with short-acting insulin.
Long-acting insulin analog	30-60 minutes	No peak	16-24 hours	Generally taken once daily.

History of insulin

In the past, pigs' and cows' pancreas were used to make early formulations of insulin, but it was hard to procure appropriate glycemic regulation due to leftover impurities ahead of the purification method [8]. The fresher, finer animal insulin is more readily handled and may reach a point of glycemic regulation identical to artificial human insulin. Statistically notable variation in hypoglycemia between human and animal insulin also seems comparable. The objective of insulin replacement therapy is always to replicate natural insulin production and avoid causing severe hypoglycemic levels. There are several insulin formulations available, each with a distinct spectrum of activity attainable to accomplish the same: insulin analogs that respond quickly (around three hours), neutral protamine, soluble insulin Hagedorn (NPH) insulin that work for 12 to 18 hours, long-running insulin (12-18 hours), and the Lente insulin (12-24 hours). The skin should be pinched to reduce the risk of muscle injection when giving insulin, and the fold should remain for 5 to 10 seconds after the injection has been given perpendicular to the skin. It would be best if you injected at a 90-degree angle or use a short needle to avoid injecting insulin repeatedly between the skin layers. Thanks to this, you can use a right angle with no issues [9].

## Review

Insulin

Three approaches have been used to genetically engineer insulin. First, efforts were made to directly separate and purify it from the human cadaver pancreas. However, there has never been significant adequacy of human tissue to make this procedure effective in enough quantity. The "semi-synthesis" method chemically transforms swine insulin into the human insulin sequence by substituting just one amino acid variance in the target coding sequence. Human insulin therapy did not generally become accessible before the 1980s until the advent of recombinant genetic modification. The human genetic code must be inserted into the host organism cell to produce insulin, usually baker's yeast or the bacterium Escherichia coli [10].

Recommendations for insulin administration

Many physicians oversee the administration of injectable or infusion therapy to diabetic patients, but there are not many printed recommendations to assist these caregivers. The critical point in the guidelines is the prevention of intramuscular injections, particularly long-acting insulin analogs, since they can lead to a severe hypoglycemic state. Currently, short needles, such as the 4-mm pen and 5-mm needles, are secure and efficient, and cause less pain; hence, they should be among the first options for all classes of people. Lipohypertrophy is one of the standard treatment complications that affect insulin absorption. Therefore, injection or infusion should not be administered in such lesions, and appropriate site changes will be of use. Improper disposal of consumed sharp objects increases the danger of infection with blood-borne microorganisms; however, this risk can be reduced with proper guidance and training, sensible disposal methods, and use of protective equipment [11]. These guidelines were created and reviewed by 183 diabetes specialists from 54 nations in Forum for Injection Technique and Therapy: Expert Recommendations (FITTER) in Rome, Italy, in the year 2015. The new insulin administration guidelines, just published in Mayo Clinic Proceedings 2016, are by far the most recent in a line of recommendations made by international specialists [12].

Insulin delivery methods

Diabetic patients administer dosages of insulin on their own with multiple daily injections (MDIs) by using syringes, pens, as well as patches. In this approach, people with diabetes routinely inject themselves with long-lasting dosages, which can be supplemented with added fast-acting insulin dosages to regulate their blood glucose levels. Multiple injections are required throughout the day to maintain normoglycemia; hence, the method of delivery should lessen injection pain to improve patient compliance with therapy [13]. In subcutaneous continuous insulin infusion, a single subcutaneous site is used by insulin pumps to infuse insulin continuously; this site is changed, on average, every three days. The only type of insulin utilized is rapid-acting, and analog insulins have become more widespread than conventional insulin for this application [14]. Compared to MDIs or traditional continuous subcutaneous infusion of insulin, sensor-augmented pump (SAP) therapy, which integrates insulin pump therapy and real-time continuous glycaemic monitoring, has enhanced metabolic control and has lowered the incidence of hypoglycemia in patients having type I diabetes mellitus [15]. There are numerous insulin pen models and brands available in market. Most of them can be divided into reusable and disposable. A prefilled insulin cartridge is used in a disposable pen. The entire pen device is discarded after a single use. A reusable insulin cartridge is located inside the pen. When the insulin-filled cartridge is empty, we can remove it and put a new one. After each insulin injection, a new disposable needle must be used. Reusable insulin pens can be used for several years with proper maintenance [16]. Omnipod DASH insulin management system by Insulet Corporation is a pod therapy that provides a tubeless, wearable insulin pump that is impervious to water and can carry up to 200 units of insulin and provide 72 hours of continuous insulin therapy using adjustable basal rates and bolus quantity. Insulin "bolus" dosages are given during meals or for correcting high blood sugar levels, whereas basal insulin dosages help maintain your blood sugar constant over time [17]. V-Go is an insulin delivery system available only through prescription for patients with type II diabetes who need to take insulin to maintain their blood glucose levels. V-Go is a practical substitute for needles and syringes for administering insulin multiple times a day, just like a conventional insulin pump, but with one significant distinction, i.e., V-Go is a debit card sized patch that attaches to the skin, as opposed to typical pumps, which contain an insulin reservoir (a device of roughly the size of a small cellphone) that is connected to the body by tubing [18].

Advancements in insulin delivery and its future

The newer insulin, known as "smart insulin," reacts to fluctuating blood glucose levels automatically [19]. A larger or smaller quantity of insulin is released, linked to the glycemic levels in circulation. The hormone insulin, whether supplied orally or intravenously, maintains steady blood sugar levels all day, which helps eliminate carb counting, several regular injections, hypoglycemia, and high blood sugar. Todd Zio, an Massachusetts Institute of Technology expert, launched a firm named SmartCells Inc. in 2003, quickly receiving support from Juvenile Diabetes Research Foundation as it attempted to create GRI (glucose-responsive insulin) [20]. This effort was one of the initial intelligent insulin efforts. With more money available recently, more groups are experimenting with ways to distribute intelligent insulin molecules, often made to circulate in the bloodstream longer than conventional insulin [21]. For many years, scientists in North Carolina have been developing an intelligent insulin patch. Researchers said in 2015 that this patching, worn on the body's exterior, utilizes a network of tiny needles for detecting high blood levels of glucose and provides the right amount of insulin. A year later, the patch was improved to include living beta-cells, which can stabilize increasing blood sugar levels for about 10 hours at a stretch. There is no chance that the body's immune system of patients with type I diabetes will reject the beta-cells because they are confined inside the patch on the exterior of the body. Animal trials have been ongoing since about 2016; however, it will take some time before human clinical trials occur [22].

**Figure 1:**
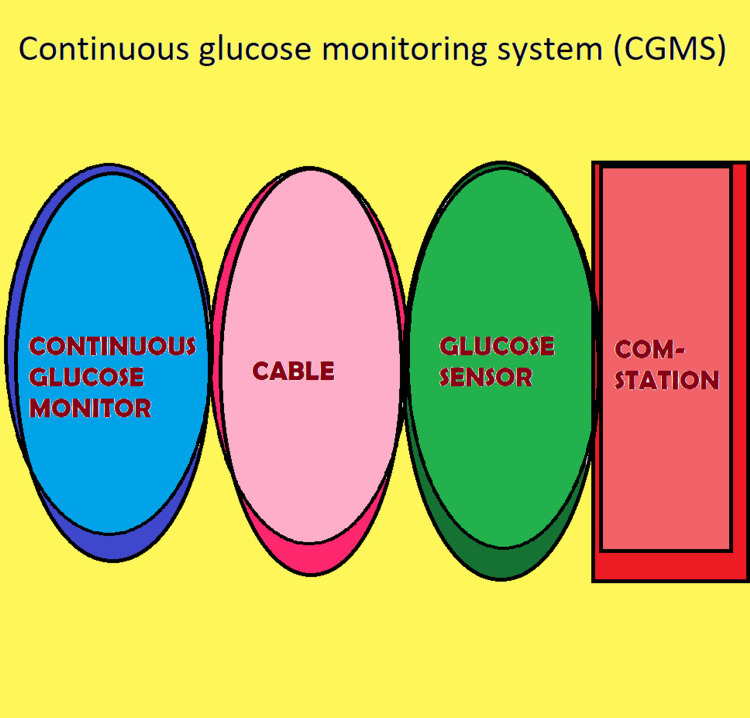
Components of continuous glucose monitoring system (CGMS) Image credit: Clipart created by the author in Paint software

Year 2021 marks the 100th anniversary of insulin's discovery. Insulin has emerged as one of the most acceptable glucose-lowering treatments for diabetes which is given to patients through syringes, pens, and pumps. But, some people feel it is inconvenient to administer insulin injections numerous times in a day. Experts at Scuola Superiore Sant'Anna and physicians at the University of Pisa are included in this movement to create closed-loop insulin delivery systems entirely internal to our body [23]. U.K. researchers have started developing an “intelligent” insulin pill. The innovative new initiative from the University of Birmingham may permit people with type 1 diabetes to get rid of routine insulin injections. When blood glucose levels rise, these intelligent capsules rest in the body and release insulin. The capsules include particles that adhere to glucose; when blood glucose levels are high, these particles in the capsules melt away, releasing the insulin. Making patients' lives better is the team's first aim, according to Dr. John Fossey, a senior lecturer in Birmingham's School of Chemistry. They are attempting to develop a mechanism to deliver more insulin if blood sugar levels are high [24]. There are two types of continuous glucose monitoring (CGM) systems: professional devices, which patients wear without being able to view glucose values until their doctor download data retrospectively during an office visit, and personal systems, which allow for both real-time and retrospective review of entire profile by patients at home, doctors in health center, or remotely (Figure 1) [25].

Regular human insulin and rapid-acting manufactured insulin are the two kinds of insulin used by the jet injector group [26]. Insulin jet injectors come with either a compressed gas cartridge or a compressed spring to create the desired pressure required to propel insulin through the jet injector into the skin. Compressed springs are used more often, and these gadgets are light, compact, durable, and affordable. The jet is loaded by filling its adapter with insulin, and once it is loaded, the gauge is set according to the calibrated insulin dosage. The device is placed against the skin, usually in the fat-rich part. The stomach, the anterior aspect or side of the thigh, and the upper or outer portion of your buttocks can be suitable locations [27]. Insulin is administered with the help of the InsuJetTM system, which was created for people with diabetes. The device's essential component is its innovative, needle-free nozzle. A very narrow stream of insulin that is easily penetrated via the skin is produced by pressing insulin through the nozzle aperture. The insulin then spreads uniformly in the subcutaneous tissue layer by following the path of least resistance. The use of jet injectors to give various live and inactivated vaccinations for viral and bacterial infections has been documented to be effective and safe [28].

Novel concepts

Discovery of Foxo

Identifying a family of insulin-responsive transcriptional proteins called Foxo proteins in nematodes marked a turning point in insulin action research. As claimed by the American Diabetes Association, the capacity of insulin to simultaneously regulate numerous genes through a transcription factor provides the best explanation for its integrated effects on various cellular activities. Although the impact of glucagon and insulin on the expression of genes was well understood, their metabolic outcome was not thoroughly understood until it became clear that diabetes could be reversed by inhibiting Foxo1 [29]. Additionally, Fox's capacity to connect a standard signal (Akt phosphorylation) to various transcriptional targets across multiple cell types is crucial for diversifying insulin signaling in different organs. It has made it possible for the two critical characteristics of diabetes (insulin resistance and pancreatic-cell dysfunction) to be combined under a single Foxo-dependent methodology [30].

Inceptor

Insulin resistance beta-cells of the pancreas lead to overt diabetes in rats; hence, the treatment that sensitizes beta-cells to insulin may shield diabetic patients from beta-cell failure. Experts have identified an inhibitor of the insulin receptor, i.e., INSR and IGF1 receptor (IGF1R) signaling in beta-cells of rats, named insulin inhibitory receptor (inceptor; encoded by the Iir gene). Inceptor has a cysteine-rich region similar to INSR and IGF1R and a mannose 6-phosphate receptor region (found in the IGF2 receptor-IGF2R). Rats deficient in the receptor inceptor present hyperinsulinemia and hypoglycemia and live only for a bit after birth [31]. Hence, we can conclude that Inceptor reduces insulin's effect and acts against its signaling (countereffect) in beta-cells to control glycemic levels [32].

Ultrastable Insulin

Weiss (Indiana University School of Medicine) believes that the development that might occur the quickest relates to “ultrastable insulins” to eliminate the requirement of cold chain. Now, we must prevent insulin from being revealed, even briefly, to temperatures over 30-35 degrees Celsius. Delivering insulin internationally and globally would be made possible by making it less expensive and its current distribution infrastructure less complicated, which hinders the treatment of the diabetes pandemic in underdeveloped nations [33].

This Might be the End of Insulin

We are getting closer to a cure for diabetes (which was unavailable to date) because of new research. Diabetes treatment was introduced a century ago. Five experts (University of Alberta) aim to eradicate insulin treatments. If this research is successful, insulin might gradually become obsolete with the passage of time. If pushed in the appropriate direction, stem cells can differentiate into any cell. Shapiro and his team are trying to genetically modify a person's biological blood cells to transform them into stem cells and then reprogram them to form insulin-producing islet cells. The islets will then be implanted into the same person's liver, and insulin will be produced there. The "super-liver" will take over the routine duties of the pancreas. There is no requirement for the anti-rejection medications that are given along with conventional transplantation procedures since the donor and the receiver are the same person [34]. But, if the donor and receiver are not the same people, then anti-rejection medications will be needed, which can raise the chance of malignant changes and renal deterioration [35].

## Conclusions

Effective glycemic management is crucial due to the high rates of morbidity and death caused by diabetes and the high expenses of treating it. Traditional syringe/vial insulin administration is accompanied by patient and clinician hurdles, including psychological insulin resistance, patients' anxiety about the consequences and harmful effects of insulin, and necessary dietary modifications or restrictions. Despite research showing that many type 2 diabetic patients cannot maintain their blood sugar levels with just oral medication, some doctors are still hesitant to start insulin treatment. Over the past several years, improvements in insulin delivery have been concentrated on enhancing patient convenience and glycemic control. The more recent insulin delivery methods include transdermal patches, inhalable devices, continuous subcutaneous insulin infusion pumps, insulin pens, and insulin injection ports.
